# A new kinematic dataset of lower limbs action for balance testing

**DOI:** 10.1038/s41597-023-02105-2

**Published:** 2023-04-14

**Authors:** Anqi Dong, Fei Wang, ZhenYu Shuai, Kaiyu Zhang, Dexing Qian, Yinsheng Tian

**Affiliations:** Beijing Sport University, Beijing, China

**Keywords:** Data acquisition, Classification and taxonomy

## Abstract

Balance is a common performance but nevertheless an essential part of performance analysis investigations in ski. Many skier pay attention to the training of balance ability in training. Inertial Measurement Unit, as a kind of Multiplex-type human motion capture system, is widely used because of its humanized human-computer interaction design, low energy consumption and more freedom provided by the environment. The purpose of this research is to use sensor to establish a kinematics dataset of balance test tasks extracted from skis to help quantify skier’ balance ability. Perception Neuron Studio motion capture device is used in present. The dataset contains a total of 20 participants’ data (half male) of the motion and sensor data, which is collected at a 100 Hz sampling frequency. To our knowledge, this dataset is the only one that uses a BOSU ball in the balance test. We hope that this dataset will contribute to multiple fields of cross-technology integration in physical training and functional testing, including big-data analysis, sports equipment design and sports biomechanical analysis.

## Background & Summary

Balance is evaluation element for the assessment of motion performance. In various biomechanical analyses, the movements of athletes are usually completed by multiple joints and muscle groups according to a certain time sequence, which is called Kinematic chain^1–3^.

Kinematic chain analysis is one of the keys in biomechanics analysis. High-speed infrared motion capture systems to track three-dimensional motion represent a baseline method used in scientific research;^4,5^ These are highly accurate but expensive and are thus often limited to being used at experimental sites. Most biomechanical research with data collection is limited to the triaxial variables of joints in anatomy, which makes it difficult to use sensor data analysis methods, such as machine learning or deep learning, for accurate analysis^6–8^. In now, wearable devices based on the combination of inertial sensing technology and wireless communication are gradually being used in the performance analysis of many sports events because of their affordability, usability, and ease of learning^9,10^. This combination is used for sports rehabilitation, the selection of athletes, and training performance analysis^11–16^.

Karl Mülly put forward the importance of balance in alpine skiing as early as 1933^17,18^. The article points out that the difference between skiing balance and daily activity balance which is in skiing balance, athletes should adjust the height of the centre of gravity according to different techniques to complete the action, especially with the advancement of ski techniques such as carving, skiers need a strong core force to control the roll edge change and front and back balance, and accurately cut the skis to achieve the best sports performance^18^. Through Staniszewski’s^7^ research also can be determined that good balance ability has a positive impact on skiing, regardless of skiers’ technical level^7^. At present, there are few literatures about balance research in skiing, and the test methods for evaluating balance do not all have enough differentiation ability. Therefore, it is helpful to improve the specific balance ability of skiers by searching for test actions from the perspective of skiers’ balance training.

In the research on the balance ability of alpine skiing, Vjekoslav combined with the BOSU ball and inertial measurement unit in the laboratory for the first time, and found that in alpine skiing, the balance ability of athletes is different due to various sports grades. And also proposed to use the BOSU ball as an auxiliary tool for balance training, which provided the possibility of training static and dynamic balance at the same time, that is, to maintain a stable position on an unstable surface, balance training on the BOSU ball is desirable^19^. In recent years, the application of wearable sensors in the field of sports has gradually become mature. The Inertial Measurement Unit(IMU) motion capture system have been used in the performance analysis of many sports events, meeting the needs of football, rugby, canoeing, skiing and other training^20^.

Among the many sensor companies, the IMU motion capture system represented by Noitom’s Perception Neuron Studio(PNS) integrates a three-bit acceleration sensor, a three-axis gyroscope, and a three-axis magnetometer, which can obtain nine degrees of freedom data; it combines machine learning and signal processing visualization technology, and its application scenarios are recognized by a wide range of sports researchers to help athletes achieve better performance in Olympic events such as figure skating, fencing, and slalom kayaking^21–23^. As a result of the Noitom’s PNS is highly sensitive to acceleration parameters, the recognition accuracy of bending/extension (FE), lateral bending (LB) and axial rotation (AR) in joint motion is better than that of other IMU sensors.

The combination of an inertial motion capture system and an infrared motion capture system is a conventional method for collecting data to promote the scientific development of sports training^5,20,21^. On the other hand, the related theories and methods of intelligent equipment combined with balance training are still in the exploratory stage, and there is a lack of public kinematics datasets on various evaluation dimensions for engineers to optimize algorithms. This project starts with the balance ability in sports, use the PNS system, whereby human actions are used in function testing to establish a dataset reflecting the balance level; This data will help facilitate future research on analysis in sport performance.

## Methods

### Preparation phase

#### Experiment design

Kinematic data were collected based on the basic concept of balance and previous research^24,25^, and we developed an improved balance test action through literature and scales. Based on the Delphi method, we conducted two rounds of investigations with six experts, and finally selected three typical actions.

During these movements, the experimental process is that the participants wear legging and wore sneakers without air cushion. The participants wear sensors on the corresponding body parts, and the PNS system selected the lower body acquisition mode, and. Before the acquisition, we will check the number of sensors, sensor initialization, signal quality, and posture calibration that ensure the device will not be put into use until it reaches the acquisition standard. The participant starts with standing. When participants are ready and we say “start”, Axis Neuron Studio software records motion data at the same time. During the test, we can observe the signal strength in real time. When the signal is poor, we will adjust it in time to ensure the integrity and validity of the data, and then continue the test. They required to complete the following actions(see Table 1)^24,26–30^.

**Table 1:** Designation of experimental tasks and measurements.

Activity	Number/Time	Items
1. Stand straight	5	Balance scale test action
2. Gait	5	Common balance test actions
3. Squat	5	Common balance test actions
4. BOSU ball Squat	10	Athletes’ common balance training movements^7,17–19^

Describes test tasks.

**Fig. 1:**
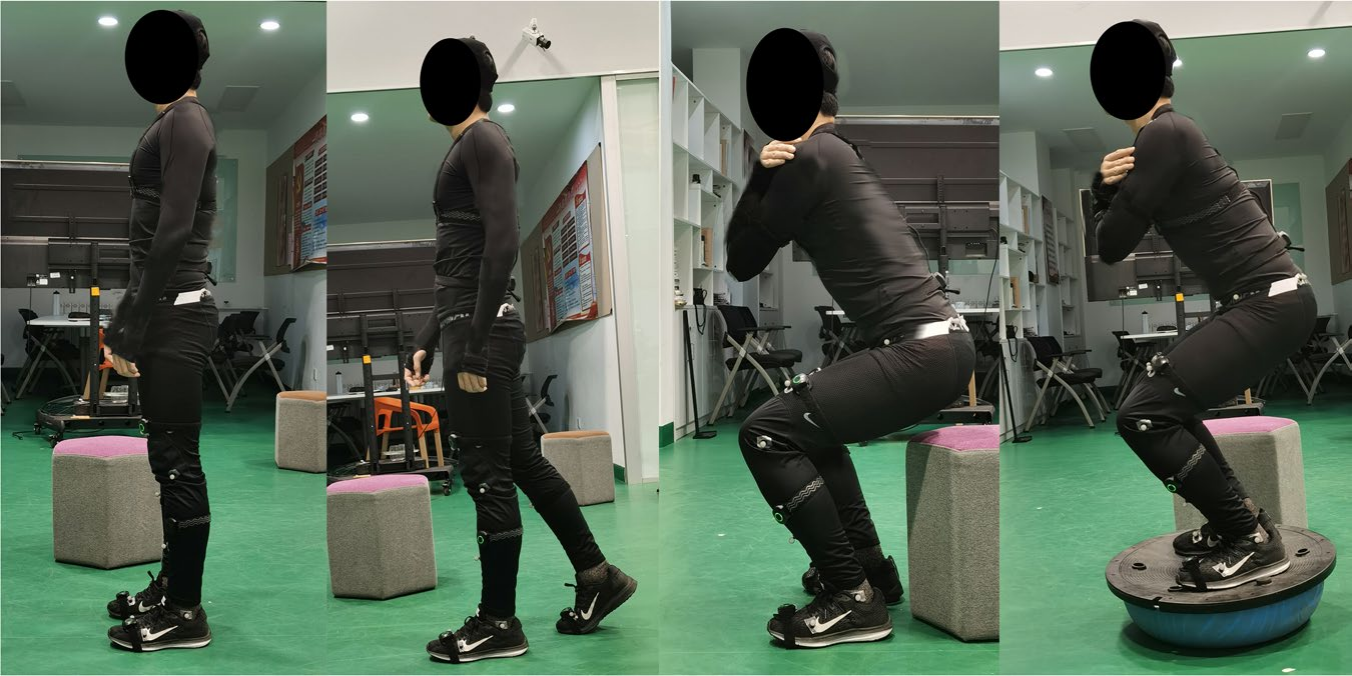
Lower limb task. The order is Stand straight, Gait, Squat, BOSU ball squat.

(1) Stand straight: this is static standing, with arms down and palms facing the body. Feet are positioned approximately the same distance as the participant’s hip width and feet are parallel to each other.

(2) Gait: fixing the number of steps the participants walk, ours each walked six steps uniformly. Step length should be adjusted according to the participant’s habits to prevent them from leaving the PNS reception range.

(3) Squat: place a 45 cm tall base behind the participant’s knees; this prevents them from squatting below this depth. This standardized method is crucial to prevent reflection markers from being blocked during a lower squat.

(4) BOSU ball squat: The standardization of the BOSU squat is mainly from the perspective of safety, so that the participants can maintain a consistent squat depth as far as possible under the condition of ensuring safety(see Fig. 1).

#### Equipment and environment

**Fig. 2:**
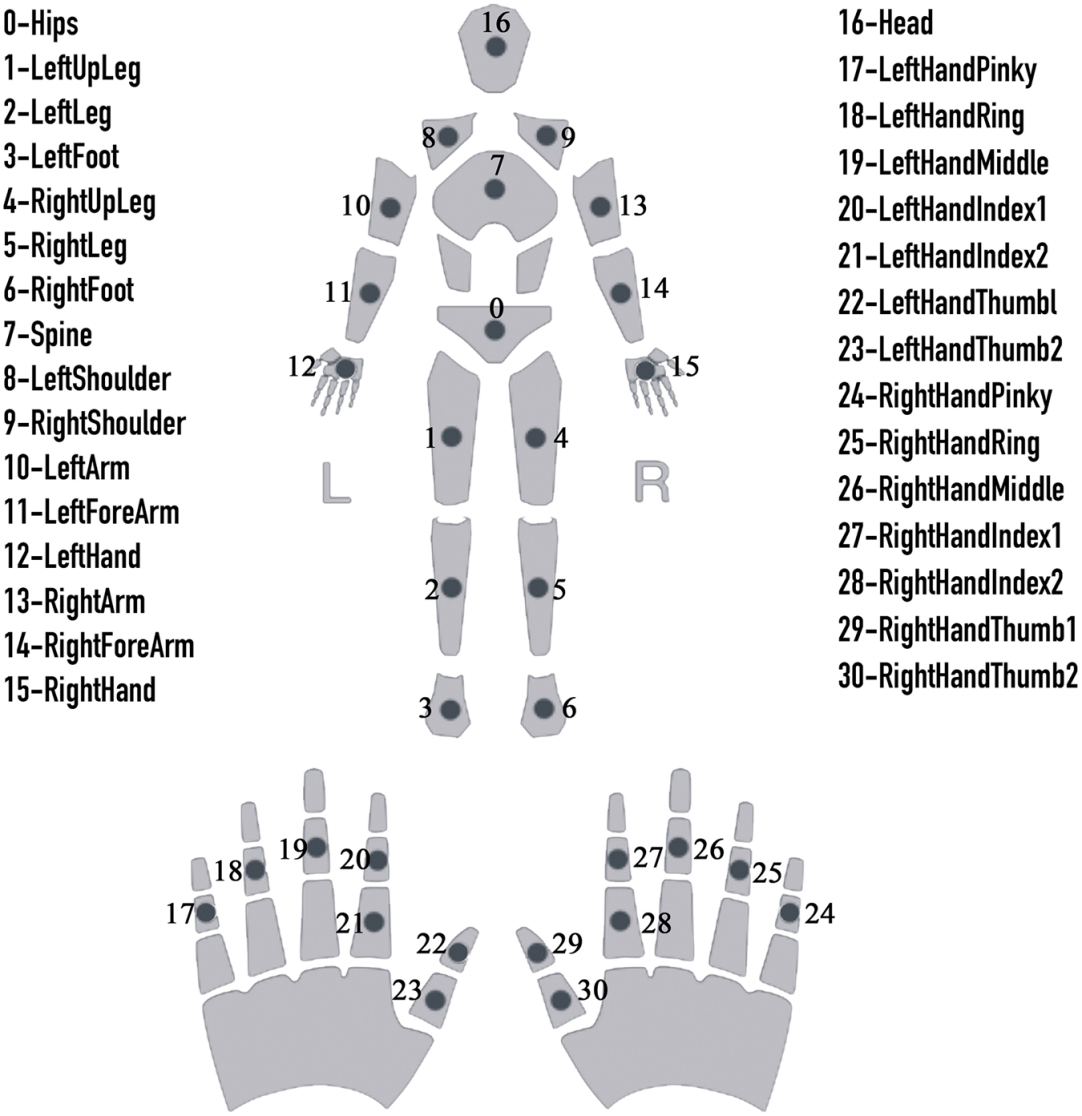
Skeletal structure. The approximate anatomical position and corresponding description of 72 nodes are given. This experiment uses 0–6 nodes of lower limbs are shown in the rectangles.

Data and signal acquisition equipment are mainly used to collect three-dimensional motion information from the movement of the human body. The motion capture system is Perception Neuron Studio (Noitom Perception Neuron Studio, Noitom Technology Ltd., Beijing, China). This system is responsible for collecting lower limb motion signals, which can be real-time transmission of acceleration (Acce), angular velocity (Gyro), quaternion (Quat), velocity (Velo), and position (Posi). In this experiment, the sampling rate of the data acquisition system is 100 Hz, Perception Neuron Studio uses ultra-high precision aerospace-grade sensor calibration, improves the measurement range of the sensor and the parameters of the sensor are show into Table 2 (see Table 2). The collection environment of the device requires the participant to wear seven sensors, as shown in (see Fig. 2), for the sensor wearing position map.

**Table 2:** Sensor parameters.

Parameters	
Sensor Size	43 mm × 33 mm × 20 mm
Sensor Weight to mass	15.8 g
Gyroscope range	±2000 dps
Acceleration metering	±32 g
Minimum Resolution	0.02 degree
Static Accuracy	Roll 0.7, Pitch 0.7, Yaw 2
Data Acquisition system	100 Hz

In order to reduce the influence of strong magnetic field environment on the output data, in the experiment that keep the field within 1–3 meters of the field away from strong magnetic equipment, and before the experiment starts, the equipment is open the anti-mag node. After the experiment, the PNS acquisition software was used to correct the data, and finally the data was uploaded to the database (see Fig. 6). In other published studies, we also experimented with participants using OptiTrack (OptiTrace, Norkov Technology Co., Ltd., China) infrared cameras to ensure data reliability^24^.

On the ferromagnetic problem interference, the tiny interference of ferromagnetic problem is certainly inevitable. However, during the experiment, the following experimental requirements can minimize the ferromagnetic problem. Firstly, the laboratory environment is highly demagnetized during the conduct of the experiment. Except for the experimental equipment, any electronic equipment is turned off and removed from the laboratory. Second, the PNS equipment used in this experiment is highly resistant to magnetization, and the data it goes to is very little affected by magnetic interference^25^.

#### Participants

A total of 20 college students passed the screening for motor function and became participants in our study. Among them, there were 10 males and 10 females with a mean age of 25.35 years and a standard deviation of 2.35. Motor function screening requires the participants to perform routine functional exercise tests to ensure that the participants have no obvious movement disorders, such as severe sports injuries, joint inflammation, or balance defects. None of the participants in our study reported any known movement disorders or other health problems that could affect mobility. The study was conducted in accordance with the Declaration of Helsinki. Before the experiment, participants were informed of the contents of the experiment, and each participant voluntarily signed an informed consent form and agreed to participate in the experiment. The body size of the participants is presented.(see Table 3).

**Table 3:** Demographic information of the participants.

participant Code	Gender	Age (years)	Height (cm)	Weight to mass (kg)	Thigh length (cm)	Shank length (cm)
P1	F	23	173	60.5	45	41
P2	F	28	163.5	47.5	43	37
P3	F	29	165.3	54.3	45	38
P4	F	28	165.1	60.5	42	38
P5	F	28	170	55.1	47	40
P6	F	24	172	62.2	41	37
P7	M	20	173.5	62	42	40
P8	M	23	184	80	49	43
P9	M	26	192	88	47	41
P10	M	24	165	64	37	37
P11	F	26	164	50.1	35	47
P12	M	28	185	83	45	50
P13	F	20	163	45	46	38
P14	M	25	174	71	41	41
P15	M	26	176	70	42	41
P16	M	29	177	77	42	41
P17	M	25	179	78	45	41
P18	F	21	153	58	40	37
P19	F	26	163.5	52	39	43
P20	M	28	179	77	40	38

Here P represents the participant, and the number following is the order in which the participants performed the experiment. Before the experiment, participants were informed of the contents of the experiment, and each participant voluntarily signed an informed consent form and agreed to participate in the experiment.

### Recording phase

**Fig. 3:**
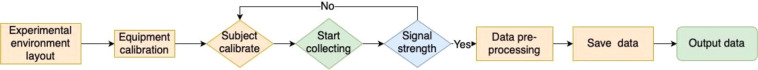
Experimental flowchart.

The participants wear tights with sensors on the corresponding body parts, and the system selects the lower body collection mode. Before the collection, we evaluate the equipment based on the number of sensors, sensor initialization, signal quality, and sensor attitude; the equipment is used only when the collection standards are reached. Because the surrounding environment affects the magnetization, we recommend using four consecutive static postures to perform a calibration of the acquisition device on the Axis Neuron Studio software (https://neuronmocap.com/perception-neuron-studio-system). To prevent the participants from misunderstanding the actions, they should be given thorough explanations and trained on the actions before the experiment. Each participant should start in a standing posture, and for each action, asked to perform the action tests. When the participant is ready, we tell them to begin and the Axis Neuron Studio software records the exercise data as the exercise is being performed. During the test period, the signal strength should be observed in real time. When the signal is poor, it is important to pause and make timely adjustments to ensure the integrity and reliability of the data before continuing the test(see Fig. 3).

## Data Records

We chose to export two different data formats: inertial data and action flow data.

The inertial data is divided into four parts per record:

1. The first field indicates the name of the body part, such as Hips, SpineBottom, etc. There are 7 body parts in this study.
2. The second field shows the feature type, which can be “Sensor”, “Joint”, or “Bone.”
3. The third section indicates the signal type: acceleration (Acce), angular velocity (Gyro), quaternion (Quat), velocity (Velo), and position (Posi). It also includes the lost packet mark, Lost, for when the signal quality is poor.
4. The fourth field contains the specific component values, including X, Y, Z components, of which the quaternion has an additional W component.

The action flow data provides human anatomy information. It can support most 3D animation production software, including 3dMax, Unity, and Maya. The action stream data format is divided into two parts:

1. Size: defines the size of the main bones of the body in cm.
2. Motion: defines the number of frames, frame rate, and the rotation angle of each joint in each frame.

The dataset is available at Figshare (10.6084/m9.figshare.20579541.v1)^26,27^. Please refer to the Code Availability for the related code. This data provides a set of motion flow data described in motion anatomy terms, the content of the document in this format is converted from a BVH file. And in anatomy, it is defined and described in terms of the way the individual bones of the human body move.(see Table 4, Fig. 5). The BVH files to calculate durations borrowed from a publicly available dataset^28^.

**Table 4:** BVH-formatted definitions of joint motion.

Joint	Motion	Anthropology	Explanation
Hip	Flexion/Extension	Thigh lift forward for flexion (+), backward for (−)	Rotation in the sagittal plane around the frontal axis
	Axial rotation	Right thigh: Counterclockwise rotation from proximal to distal is internal rotation (+), and opposite is external rotation (−). The left side is opposite to the right side	Rotation in the horizontal plane around the vertical axis
	Lateral bend	Right thigh, Left thigh: both rotated medially for adduction (+) and opposite for abduction (−)	Rotation around the sagittal axis in the frontal plane
Knee	Flexion/Extension	The knee is bent backward for flexion (+), and opposite for extension (−)	Rotation in the sagittal plane around the frontal axis
	Axial rotation	Right thigh: counterclockwise rotation from the proximal end to the distal end is internal rotation (+), and opposite is external rotation (−). Left thigh: opposite to right thigh	Rotation in the horizontal plane around the vertical axis
	Lateral bend	Left/Right: knee-adduction (+), knee-abduction (−)	Rotation around the sagittal axis in the frontal plane
Ankle	Flexion/Extension	The back of the foot is pressed down and straightened for flexion (+), and opposite for extension (−)	Rotation in the sagittal plane around the frontal axis
	Axial rotation	Right foot: counterclockwise rotation from the near end to the far end for internal rotation (+), and opposite for external rotation (−); left foot: the opposite of the right foot	Rotation in the horizontal plane around the vertical axis
	Lateral bend	Inward turn: the direction of the outside of the foot along the ground (+); opposite for outward turn (−)	Rotation around the sagittal axis in the frontal plane

**Fig. 4:**
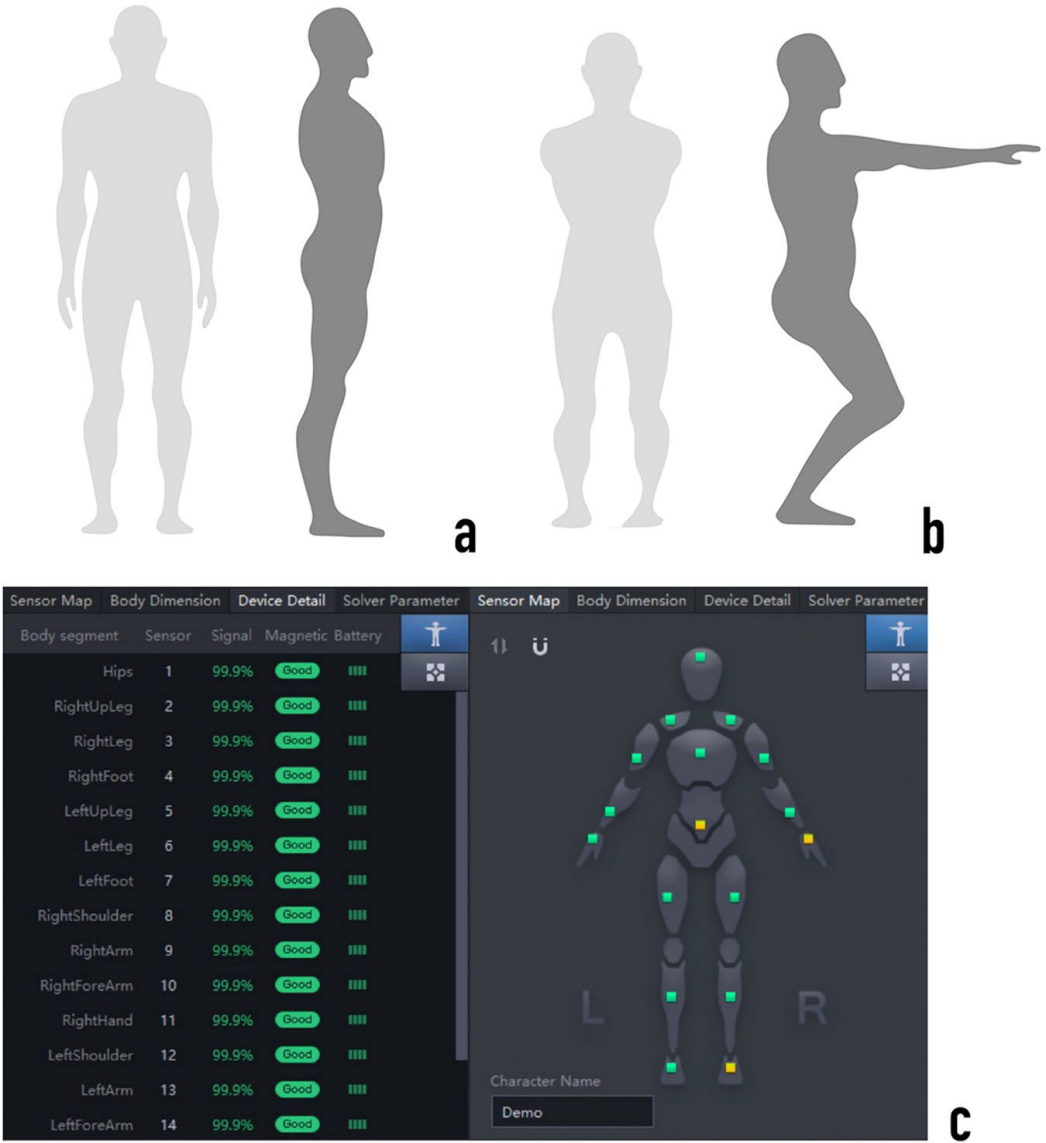
Two-step calibration procedure. (**a**) A pose: Stand straight, arms facing down with palms facing the body. (**b**) S pose: Crouch down with feet flat on the ground while maintaining feet and legs parallel to each other. Extend arms forward with palms facing down. (**c**) Sensor signal strength: Green is good, yellow is medium, and red is poor. Black means there is no sensor linked.

**Fig. 5:**
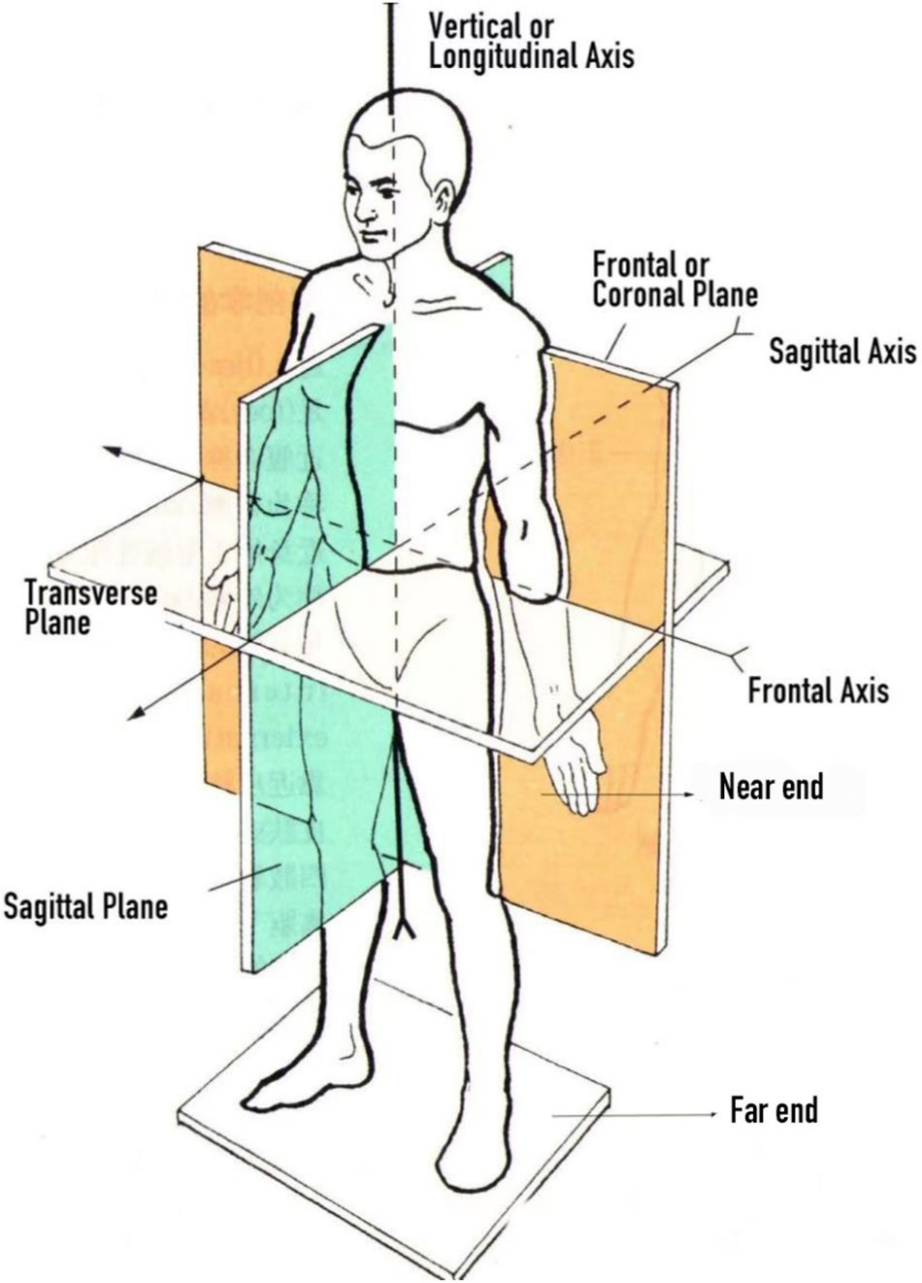
Anatomical position: The body is upright, legs together, and directed forwards. The palms are turned forward, with the thumbs laterally.

## Technical Validation

### Validity

Movement of the participants’ lower limbs was evaluated by three-dimensional analysis of the functional movement of the hips, knees, and ankles^24,29,30^. The multiple correlation coefficient (CMC) was used to compare the range of motion (ROM) with Optitrack. By calculating the PNS and OptiTrack system in the test tasks, the posture angles of all joints have excellent similarity, and the lateral and frontal angles of all joints during squatting and walking have good similarity^24^. It shows that the experimental data have good validity.

On this motion plane, the joint angle waveform obtained by the PNS system was similar to that obtained by the Optitrack system. This indicates that the proposed equipment has good reliability. θ_f_ is the angle at time point ƒ that has been measured with the method M. M = 2, as there are two methods—PNS and OptiTrack—and F is the number of time points. θ_f_ is the mean angle at the time point. ƒ is between the angles measured by the two systems.

$$CMC=\sqrt{1-\frac{{\sum }_{m=1}^{M}\;{\sum }_{f=1}^{F}{({\theta }_{mf}-\bar{\theta }{}_{f})}^{2}{\div}F(M-1)}{{\sum }_{m=1}^{M}\;{\sum }_{f=1}^{F}{({\theta }_{mf}-\bar{\theta })}^{2}{\div}(MF-1)}}$$

### Calibration

As mentioned in the recording phase, a four-step calibration procedure was used to calibrate the motion capture system prior to recording and again as needed. After all the sensors were calibrated, the initial posture of the participant was consistent with the posture of the model in the software, as shown in (see Fig. 4a): facing forward, arms hanging naturally at the participant’s sides. We kept all models in the central region of the software’s sensor area to keep the models’ sensor locations stable. To assess the quality of the calibration, we extracted the X, Y, and Z positions of the center of mass in the last three frames of the subject’s stand straight while in the same position (see Fig. 7).

**Fig. 6:**
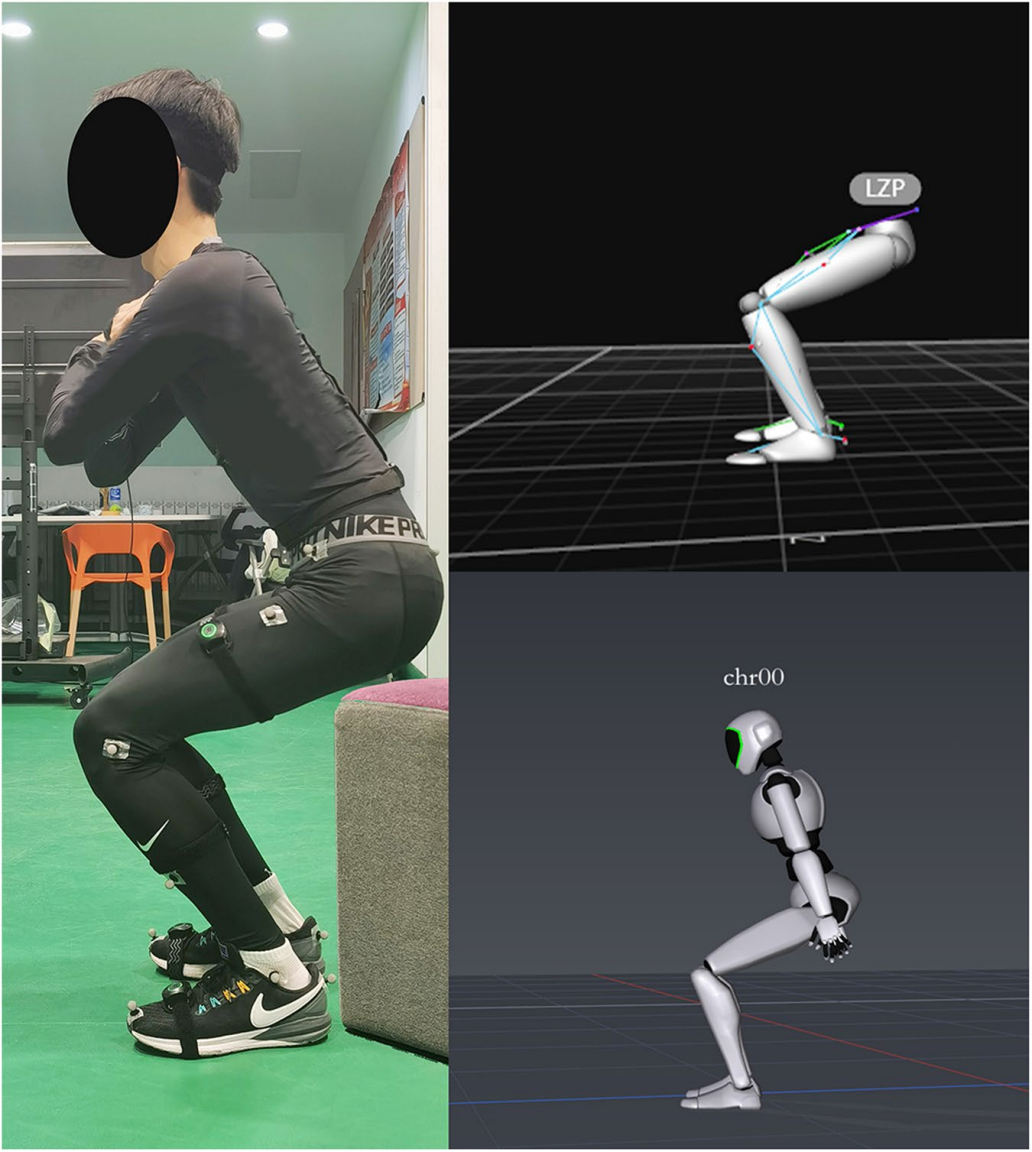
Lower limb task, and comparison chart of OptiTrack and PNS.

**Fig. 7:**
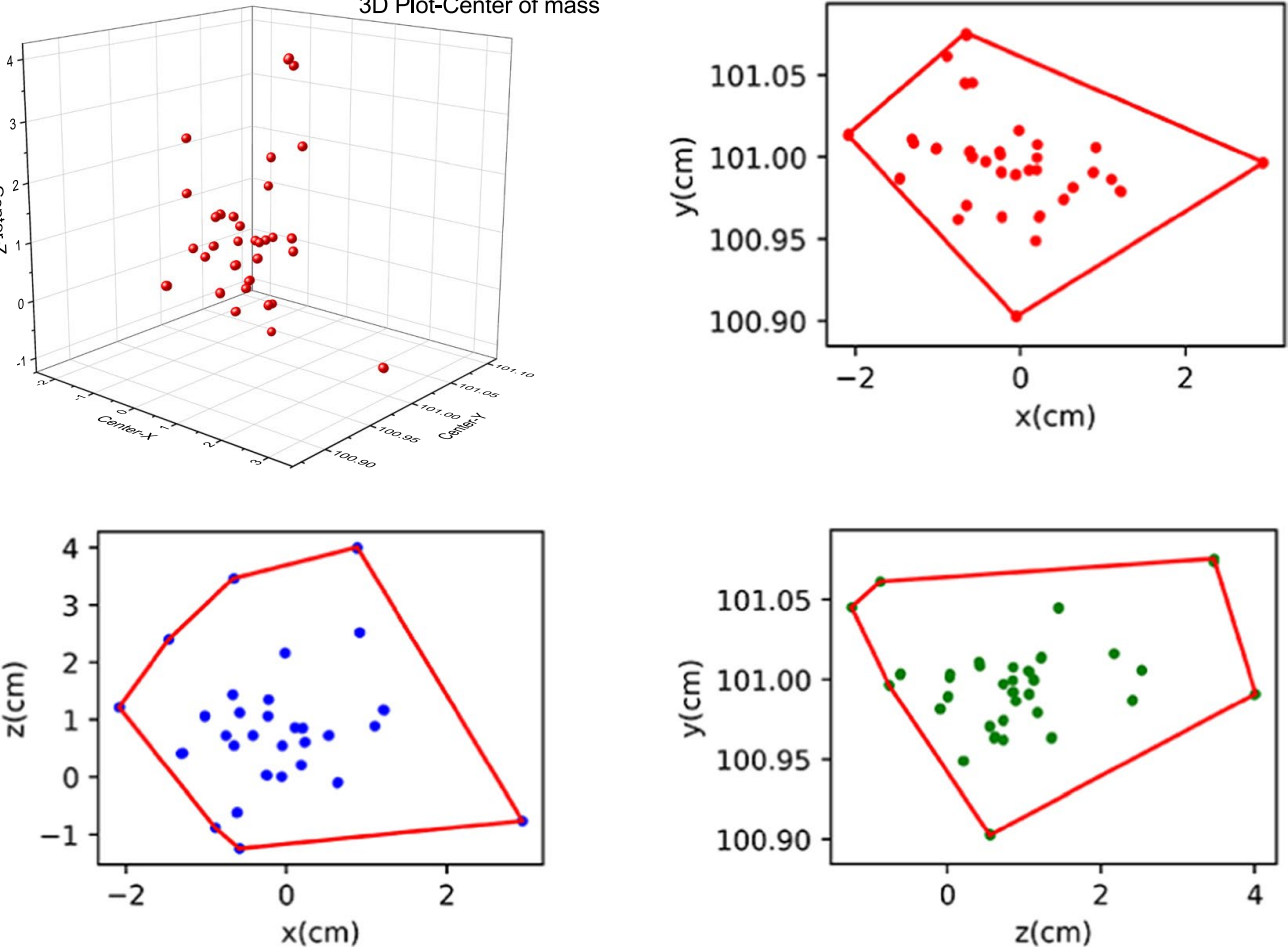
(**a**) The 3D scatter of the mass centre of the last three frames. (**b**) The convex hull graph distribution of projection on the XY axis. (**c**) The convex hull graph distribution of projection on the XZ axis. (**d**) The convex hull graph distribution of projection on the YZ axis.

After the calibration procedure, In Fig. 7, the projection deviation is concentrated at 5 cm **in** convex hull, This indicates the initial spatial position of the participants across task are relatively centralized and consistent, reflecting a good calibration quality in the present study^20^.

## Usage Notes

BVH data can be imported directly into 3ds Max or Unity, or into other applications that support it with the appropriate plug-ins. It can be processed using the Python parsing function of the PNS (https://bvh.noitom.cn, custom code cannot be shared because parsing the site is private.), which has the added benefit of defining and describing the various bones in the human body. However, researchers should be aware of several anomalies in the research data: For example, because of the influence of various factors, there are abnormal values in the data which led to missing frames; therefore, in the experimental data processing, we chose to use the PNS software to fill in these missing frames first. Researchers should also pay attention to the lack of markers in these filled frames, which may affect the research results. In addition, we will further study the relationship between action recognition analysis and objective posture evaluation. In the field of sports and technology, we will explore the use of complex and accurate sensing equipment to visualize technical actions in the field of sports and technology to solve the problem of pain points in data analysis in the field of physical training^31–36^.

## Data Availability

The data processing procedures that we perform on the dataset are those mentioned above. 1. The Python code used to extract the frame number of CSV files to be found at 10.6084/m9.figshare.20579541.v1^27^ (Csv and Bvh are the raw data and angle is the corresponding parsed data part. The csv file initials have been updated to the participant experiment sequence number.) 2. Web-based download: the BVH files to calculate durations can be found at https://physionet.org/content/kinematic-actors-emotions/2.1.0/.
